# Delivery of Mesenchymal Stem Cells from Gelatin–Alginate Hydrogels to Stomach Lumen for Treatment of Gastroparesis

**DOI:** 10.3390/bioengineering5010012

**Published:** 2018-02-07

**Authors:** Binata Joddar, Nishat Tasnim, Vikram Thakur, Alok Kumar, Richard W. McCallum, Munmun Chattopadhyay

**Affiliations:** 1 Inspired Materials & Stem-Cell Based Tissue Engineering Laboratory (IMSTEL), Department of Metallurgical, Materials and Biomedical Engineering, University of Texas at El Paso, 500 W University Avenue, El Paso, TX 79968, USA; ntasnim@miners.utep.edu (N.T.); akumar3@utep.edu (A.K.); 2 Border Biomedical Research Center, University of Texas at El Paso, 500 W University Avenue, El Paso, TX 79968, USA; 3 Department of Biomedical Sciences, Center of Emphasis in Diabetes and Metabolism, Texas Tech University Health Sciences Center, 5001 El Paso Drive, El Paso, TX 79905, USA; vikram.thakur@ttuhsc.edu; 4 Department of Internal Medicine, Paul L. Foster School of Medicine, Texas Tech University Health Sciences Center, 4800 Alberta Avenue, El Paso, TX 79905, USA; richard.mccallum@ttuhsc.edu

**Keywords:** tissue engineering, lumen, stem cells, interstitial cells of Cajal, hydrogel scaffolds

## Abstract

Gastroparesis (GP) is associated with depletion of interstitial cells of Cajal (ICCs) and enteric neurons, which leads to pyloric dysfunction followed by severe nausea, vomiting and delayed gastric emptying. Regenerating these fundamental structures with mesenchymal stem cell (MSC) therapy would be helpful to restore gastric function in GP. MSCs have been successfully used in animal models of other gastrointestinal (GI) diseases, including colitis. However, no study has been performed with these cells on GP animals. In this study, we explored whether mouse MSCs can be delivered from a hydrogel scaffold to the luminal surfaces of mice stomach explants. Mouse MSCs were seeded atop alginate–gelatin, coated with poly-l-lysine. These cell–gel constructs were placed atop stomach explants facing the luminal side. MSCs grew uniformly all across the gel surface within 48 h. When placed atop the lumen of the stomach, MSCs migrated from the gels to the tissues, as confirmed by positive staining with vimentin and *N*-cadherin. Thus, the feasibility of transplanting a cell–gel construct to deliver stem cells in the stomach wall was successfully shown in a mice stomach explant model, thereby making a significant advance towards envisioning the transplantation of an entire tissue-engineered ‘gastric patch’ or ‘microgels’ with cells and growth factors.

## 1. Introduction

Gastroparesis (GP) is a common gastrointestinal (GI) motility disorder characterized by delayed gastric emptying without any mechanical obstruction, and is known to affect almost 10 million individuals in the United States. Depletion or structural changes of interstitial cells of Cajal (ICCs) in the diseased gastric tissue [1] have been noted in studies with animal models, as well as in patients with GP. It is well known that ICCs function as the pacemakers of the GI tract, and are involved in the transmission of the neuronal signaling to the smooth muscles. Therefore, their existence in the stomach wall is of prime importance for their properties of slow-wave generation and propagation, which allow for the movement of food through the digestive canal [1]. Loss of ICCs is believed to result in conditions of gastroparesis, and may even lead to gastric cancer [2,3]. GP is also associated with the depletion of enteric neurons, including nitric oxide synthase (nNOS)-expressing neurons [4]. The depletion of nNOS results in pyloric dysfunction and delayed gastric emptying [4]. Treatment options are limited, with the most common treatment being surgical resection of the stomach or gastrectomy, however, post-gastrectomy, many patients suffer various unwanted after-effects including bloating, loss of appetite and malnutrition [1]. Regenerative stem cell therapies, based on principles of tissue engineering, have been proposed as a therapeutic possibility to restore the levels of depleted ICCs and the normal physiological functions of the stomach wall [5]. Previous studies adopted an acellular materials-based approach using collagen-based scaffolds to induce new tissue growth within the host [5,6,7], but these efforts failed to restore function to the diseased stomach wall. Other cell-based approaches were centered on building stomach–epithelium–organoid units for overcoming the difficulties of isolating and culturing gastric epithelial cells in vitro [5]. These efforts led to the development of vascularized tissue with a neo-mucosa, and also indicated the presence of a smooth muscle layer and gastric epithelium, as well as the existence of parietal cells of the stomach mucosa, post-implantation [5]. However, the isolation of stomach–epithelium–organoid units is extremely challenging [5]. We conceived an alternative, simpler and more feasible technique of delivering cells from hydrogel scaffolds to the stomach tissue lumen in vitro such that, if successful, this approach can then be translated in vivo. In this study, mouse mesenchymal stem cells (MSCs) were seeded atop an alginate–gelatin scaffold for placing on luminal surfaces of mouse stomach explants in vitro. The possibility of using bone marrow and other non-gut-derived murine MSC for in vivo immunosuppression after allogeneic transplantation is well established [8]. We hypothesized that the mouse MSCs would adhere and proliferate within the alginate–gelatin scaffold, and upon being placed atop the stomach tissue, would migrate from the gels to the actual tissue sections. The results yielded from this work will lead us to our long-term goal, to deliver MSCs or induced pluripotent stem cells (iPSCs) from a bioengineered scaffold to the host stomach wall, to help restore the depleted levels of ICCs and lead to regeneration of smooth muscle tissue leading to overall physiological improvement of the stomach wall. Regeneration of ICCs and nNOS-expressing neurons in the stomach wall would restore gastric function in GP [9]. Stem cell therapy is considered as a potential treatment for GP [10]. However, studies on this novel treatment strategy are scarce, majorly because of technological limitations, including short-term survival of the delivered cells and their insufficient adhesion and migration, as well as insufficient regeneration of the target cells, which are affected during pathological conditions [11]. MSCs have been successfully used in animal models of GI diseases including colitis, and could regenerate enteric neurons and glia [8]. However, no previous study has been performed with these cells on GP models. As ICCs arise from mesenchymal precursors [12,13], we envision that the MSCs can help regenerate the ICCs in gastric tissue, by directly differentiating into the latter [14,15,16,17], or by releasing paracrine factors that may help restore the depleted levels of ICCs [18]. Our pilot study, for the first time, will highlight the feasibility of delivering MSCs via a hydrogel scaffold to stomach tissues in vitro. In future, this cell–gel therapy can be administered via injection to the stomach/GI tract in vivo during the course of a routine endoscopy procedure [19] as microgels [20], for delivering stem cells including MSCs, iPSCs and other regenerative factors via laparoscopy, to the GP stomach lumen in the clinic.

## 2. Materials and Methods

### 2.1. Materials and Procedure for Fabrication of the Alginate–Gelatin Hydrogel

The method used for hydrogel synthesis was adopted from previous studies carried out by Wang et al. [21] and Hernandez et al. [22]. Sodium alginate (Cat. No. 218295) and type-A gelatin (Cat. No. 901771) were obtained from MP Biomedicals (Strasbourg, France). 1-Ethyl-3-(-3-dimethylaminopropyl) carbodiimide hydrochloride (EDC, Cat. No. 22980) and *N*-hydroxysuccinimide (NHS, Cat. No. 24500) were purchased from Thermo Scientific (Rockford, IL, USA). Calcium chloride (CaCl_2_, Cat. No. C79-500) and 1× phosphate-buffered saline (PBS) were purchased from Fisher Chemicals (Fair Lawn, NJ, USA). The hydrogel was prepared by the crosslinking of an alginate and gelatin mixture with 1-ethyl-3-(3-dimethylaminopropyl) carbodiimide (EDC) and *N*-hydroxy-succinimide (NHS), followed by CaCl_2_. EDC was added to activate the carboxyl groups of alginate to form active ester groups that would promote NHS bonding with alginate [22,23,24]. Briefly, in 10 mL distilled water, 200 mg of each of gelatin and sodium alginate were added and stirred (at room temperature for 15 min at 100 rpm). After this, 25 mg EDC was added (stirred at room temperature for 10 min), followed by addition of 15 mg NHS (stirring for another 5 min at room temperature) to make the gel-like mixture. For sterilization, this gel mixture was irradiated with UV light for 30 min. After sterilization, the viscous mixture was poured into a 100 mm petri dish and tapped gently to make a smooth layer with a flat surface. After this, 1 M CaCl_2_ was added on the surface of the gelatin–alginate mixture and allowed to react for 15–20 min. Then, the CaCl_2_ solution was removed from the surface of the formed gel and the petri dish with gel washed with 1× PBS, thrice.

### 2.2. Characterization and Analysis of the Hydrogel

For all characterization and material analysis, gels cast without cells were used. All samples were present in triplicate unless otherwise mentioned.

(a) X-ray diffraction (XRD) analysis—for the phase analysis using XRD, the gel samples were frozen and lyophilized prior to XRD (D8 Discover, Bruker’s diffractometer, Karlsruhe, Germany). XRD was carried out at 40 kV voltage and 40 mA current with CuKα wavelength (1.54056 Å) and 2θ ranges from 10° to 50° at a scanning rate of 3°/min with a step size of 0.1°.
(b) Fourier-transform infrared spectroscopy (FTIR)—FTIR was used to reveal information about the molecular structure of the crosslinked gel sheet. Attenuated total reflectance (ATR)–FTIR spectra of a representative gel sample were acquired using a Perkin-Elmer, Spectrum 100, Universal ATR Sampling Accessory within the range of 700–3700 cm^−1^ in transmittance mode. Spectral manipulations were performed using the spectral analysis software GRAMS/32 (Galactic Industries Corp., Salem, NH, USA). External-reflection FTIR was recorded on a Specac grazing angle accessory using an s-polarized beam at an angle of incidence of 40° and a mercury cadmium telluride (MCT/A) detector. A piranha-treated silicon wafer was used as the background.
(c) Scanning electron microscopy (SEM)—SEM was operated in secondary electron mode for the analysis of the morphology of the gel samples, as done before [25]. Samples were visualized using SEM (S-4800, Hitachi, Japan) at voltages of 8 kV. Prior to SEM, to minimize charging during observation, samples were coated using graphite spray (Electron Microscopy Sciences, Hatfield, PA, USA).
(d) Swelling and degradation—to account for the hydration parameters of the alginate–gelatin gels leading to swelling, gels were allowed to swell to equilibrium for 5 days in Simulated Gastric Fluid (Ricca Chemical, Arlington, TX, USA) at 25 °C, to identify the time point when the weight of the gels was found to be constant, or the final swelling degree was attained [25]. Disc-shaped punch-out samples (8-mm biopsy punch) were lyophilized to reveal their dry weight (*W*_0_), prior to being exposed to the aqueous media. The gels were then allowed to swell, during which time they were taken out at regular intervals of 1 day, the excess surface liquid was absorbed using blotting paper and the gels were weighed (*W*_t_). The swelling ratio (*D*_s_), or the degree of swelling, was calculated using (1), where *D*_s_ was the degree of swelling, and *W*_0_ and *W*_t_ were the weights of the samples in the dry and swollen states, respectively.
  *D*_s_ = (*W*_t_ − *W*_0_)/*W*_0_ (1)
(e) Mechanical testing—all mechanical testing and analysis was done using an ElectroForce 5100 Biodynamics Test Instrument from ElectroForce Systems (Bose Corporation, Framingham, MA, USA). For the mechanical testing, it was absolutely necessary to use gels that exhibited smooth surfaces, after being cast and crosslinked. For making samples, dog-bone-shaped gels were cut using a mold placed on the alginate–gelatin hydrogels and carefully mounted between pressure grips, as done before [25]. Mounted specimens had an estimated cross-sectional area of 5 mm and a gauge length of 15 mm. They were maintained in CaCl_2_ to prevent aging of the hydrogels. The mechanical properties of the hydrogels were evaluated by measuring stress–strain curves via uniaxial compression at the rate of 1 mm min^−1^ until they were completely fractured. The elastic modulus of each sample was calculated from the slope of the stress–strain linear curves generated. Data are expressed as the mean ± standard deviation.

### 2.3. Cell Culture and Proliferation

Mouse MSCs (strain C57BL/6 mouse mesenchymal stem cells), basal culture medium and growth supplements were obtained from Cyagen (Santa Clara, CA, USA). A green fluorescent dye (PKH67, Cat. No. MINI67) for the pre-staining of cells prior to cell culture was purchased from the Sigma-Aldrich (St. Louis, MO, USA). 1× Cell Dissociation Medium (0.25% trypsin supplemented with 2.21 mM EDTA, Cat. No. 25-053-Cl) and 1× PBS (Cat. No. K812-500) were purchased from Mediatech, Corning (Masassas, VA, USA) and Amresco (Solon, OH, USA), respectively. Prior to being used for cell culture, the alginate–gelatin scaffolds were coated using poly-l-lysine (Sigma-Aldrich, St. Louis, MI, USA) to enhance cell adhesion [26], and UV sterilized again (~15 min). For cell seeding atop the scaffolds, mouse MSCs were thawed and seeded in a 25 cm^2^ tissue culture flask containing 5 mL tissue culture medium (MUXES-90011) (obtained from Cyagen) and supplemented with 10% FBS, glutamine, penicillin–streptomycin, nonessential amino acids, Leukemia Inhibitory Factor and 2-mercaptoethanol. Passaged and stabilized mouse MSCs were trypsinized using 0.25% trypsin–EDTA and cells prestained with PKH67 as per manufacturer’s protocols. These prestained cells were centrifuged to remove the cell-suspension media and were seeded atop these scaffolds in a density of 50,000 cells/mL.

To estimate cell proliferation of the mouse MSCs, the cells (not prestained with PKH67) were pre-stained using the Cell Trace Violet (CTV) proliferation kit (Invitrogen, Carlsbad, CA, USA) using manufacturer’s protocols [27]. These prestained cells were cultured for 72 h (37 °C, 5% CO_2_) in plastic tissue culture dishes. After 72 h, cells were detached using trypsin–EDTA (0.25%, phenol red) (Thermo-Fisher, Waltham, MA, USA), extracted and processed for flow cytometry (FACS). Extracted cells were fixed and processed further for FACS (Beckman Coulter Gallios Flow Cytometer, Brea, CA, USA) using excitation and emission wavelengths of 405 and 450 nm, respectively. Negative controls included non-CTV-stained cells grown on plastic petri dishes for 72 h.

### 2.4. In-Vitro Transplants of Cell–Gel Constructs Atop Stomach Tissue

All institutional and national guidelines for the care and use of laboratory animals were followed and approved by the appropriate institutional committees at Texas Tech University Health Sciences Center (TTUHSC). C57BL/6 mice were euthanized and their stomachs were harvested. The harvested stomach was drained of its contents and cleaned with 0.9% saline wash (BD Scientific, San Jose, CA, USA). The stomach was then transferred to a cell culture dish (60 × 15 mm) containing the growth medium (DMEM 10566-016) supplemented with GlutaMax 1, 10% fetal bovine serum, 1% nonessential amino acids, 1% sodium pyruvate, 1% penicillin–streptomycin (all sourced from Gibco), 50 mg/mL gentamicin (Sigma-Aldrich, St. Louis, MI, USA) and 10 μg/mL insulin-transferrin-selenium-X (Sigma-Aldrich, St. Louis, MI, USA). The stomach explants were left overnight (24 h) in this medium [28]. In the meantime, MSCs were dispersed by trypsin–EDTA and were reseeded on the hydrogel sheet until a monolayer of MSCs was observed. A piece of the hydrogel sheet (~5–6 cm^2^) with the monolayer of MSCs was cut carefully. This piece of hydrogel was then placed onto the luminal side of the stomach. The stomach along with the hydrogel were kept incubating (37 °C, 5% CO_2_) for another 48 h. 

### 2.5. Probing the Migration of MSCs from Gels into Tissue (Immunocytochemistry)

Chemicals used in this step included an optimum cutting temperature (OCT) compound (Embedding Medium, Fisher HealthCare, Pittsburgh, PA, USA), 4% paraformaldehyde (Sigma-Aldrich, St. Louis, MI, USA) and Fluoromount G (Electron Microscopy Sciences, Fort Washington, PA, USA) for mounting the tissue sections. Antibodies used were anti-vimentin (1:400; Cell Signaling Technology Inc., Beverly, MA, USA), anti-*N*-cadherin (1:400 Cell Signaling, Danvers, MA, USA) and Alexa Fluor 594 goat anti-rabbit IgG (1:1000, Molecular Probe, Eugene, OR, USA). As a counterstain, DAPI (Vector Labs, Burlingame, CA, USA) was used. After 48 h, the medium was removed from the wells and the hydrogel sheet along with the stomach were washed once with PBS, and then embedded in the OCT compound (Embedding Medium, Fisher HealthCare, Pittsburgh, PA, USA) for frozen tissue sectioning. The tissue was cryosectioned at 5 μm and collected on gelatin-coated slides, fixed with 4% paraformaldehyde for 20 min, washed 3× with PBS, and incubated with blocking solution (PBS with 1% normal goat serum and 0.3% Triton X-100) for 1 h, then washed once. The stomach sections were then incubated with anti-vimentin (1:400; Cell Signaling, Danvers, MA, USA), anti-CD44 or anti-CD45 (1:500, Novus Biologicals, Littleton, CO, USA), anti-CD117 (1:400, Thermo-Fisher, Waltham, MA, USA), or anti-*N*-cadherin (1:400 Cell Signaling, Danvers, MA, USA) for 2 h at room temperature and washed thrice with 1× PBS followed by incubation in the secondary fluorescent antibody, Alexa Fluor 594 goat anti-rabbit IgG (1:1000, Molecular Probe, Eugene, OR, USA) for 1 h at room temperature. The specimens were then washed 3× with PBS followed by DAPI staining (1:50,000). The specimens were again washed 3× with PBS and mounted with water-based Fluoromount G (Electron Microscopy Sciences, Fort Washington, PA, USA). Digitized images of immunostained sections were captured with a Nikon NiE fluorescent microscope (Nikon Instruments Inc., Melville, NY, USA), and analyzed using the NiS elements computer-based image analysis system (Nikon). The intensity of the immunostained stem cells in the stomach tissue was determined using an image analysis software, ImageJ (National Institutes of Health, Bethesda, MD, USA). Three cross-sections of the tissues were evaluated in each case.

## 3. Results

### 3.1. Phase Identification and Chemical Characterization

**Figure 1:**
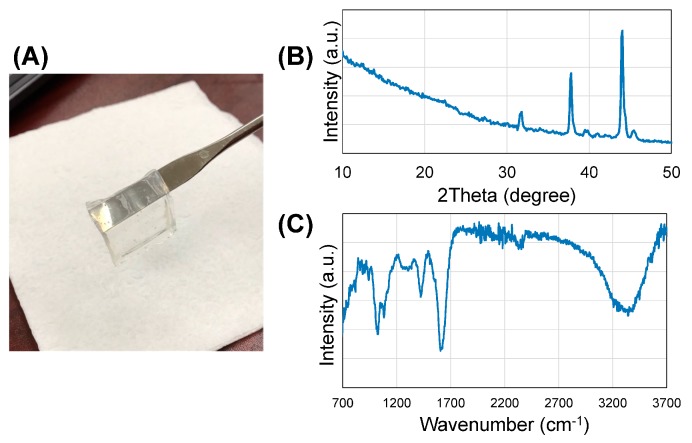
(**A**) Hydrogel sheet; (**B**) XRD spectra of the hydrogel; (**C**) FTIR spectra of the hydrogel.

The crystalline phases of the gelatin–alginate hydrogel sheet about 2–4 mm thick (Figure 1A) were determined by X-ray powder diffraction after being frozen and lyophilized. The X-ray diffraction (XRD) patterns of alginate–gelatin are shown in Figure 1B. The diffraction peak for sodium alginate at 2θ = 38.40° appeared with high intensity in the spectra, with another high-intensity peak appearing at 2θ = 43.930°, which suggested that the composition became crystalline from the semi-crystalline nature of alginate [29]. Some of the other characteristic peaks for sodium alginate were observed in the scaffold with a slight shift in 2θ values (28.96° to 31.74°, and 36.64° to 39.62°) [29]. The absence of other lower-angle diffraction peaks of alginate (at 13.570° and 22.750° [30]) and the characteristic peak of gelatin (at 2θ = 20.90° for triple-helical crystalline structure [31]) show the strong interactions between alginate and gelatin in the scaffold and more crystalline characteristics [32]. The crystalline nature indicates that the gelatin was modified with alginate after being crosslinked, which can provide better tissue culture properties for the scaffold, such as cell adhesion, hydrophilicity, increased biomechanical functionality, and biodegradation rate [33]. The components of the gelatin–alginate scaffold were determined by FTIR spectroscopy after being frozen and lyophilized. There were four characteristic peaks of the scaffold, shown in Figure 1C. The characteristic peaks appeared at 3430 (–OH stretching), 1616 (–CO– stretching), 1417 (–COOH stretching), 1092 (C–O stretching) and 1030 cm^−1^ (C–O–C stretching), which are characterized by the saccharide structure of alginate [34]. The characteristic peaks of gelatin protein structure (at 1630 and 1543 cm^−1^) assigned to the N–H stretching vibration peaks for amide I and amide II are absent in the spectra, proving the involvement of this group in the crosslinking reaction, which is in agreement with the XRD results [32]. 

### 3.2. Microstructure Imaging

**Figure 2:**
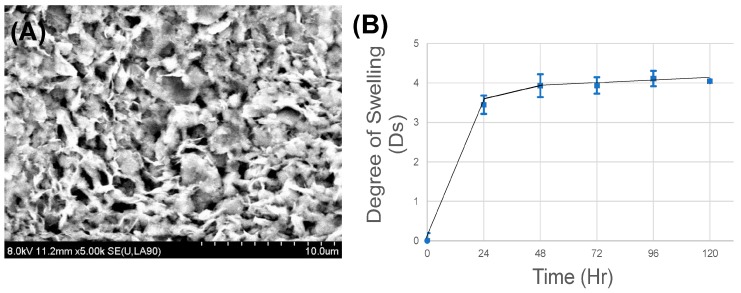
(**A**) SEM image of the hydrogel; (**B**) swelling and degradation analysis.

SEM images revealed a highly porous structure of the hydrogel with an average pore size of 1.05 ± 0.37 μm and apparent porosity of 9% (Figure 2A). The pores appeared to be homogenously networked and distributed throughout the entire structure (Figure 2A). This indicated that this structure would allow enhanced circulation of nutrients and media, and would be stable in vivo. As such, the scaffold was up to 5 mm thick resulting from the technique used for casting. We anticipate that this scaffold can be made thinner, ~200–500 μm, if we adopt 3D printing for casting these scaffolds.

### 3.3. In-Vitro Stability

Swelling analysis performed using simulated gastric fluid showed maximum swelling after 24 h, after which the gels were seen to attain equilibrium with no evidence of degradation (Figure 2B). Although the gels showed an equilibrium swelling degree of ‘4’ compared to their initial dry state, this is expected to promote loosening of the gel scaffold, thereby permitting tissue fluids to enter the scaffold and enhance the motility and delivery of cells and growth factors from within the scaffold to the host tissue. These cell–gel constructs are expected to maintain their structural fidelity when implanted in vivo for a sustainably long period of time (>120 days), even when exposed to the harsh acidic environment of the stomach.

### 3.4. Mechanical Stiffness

**Figure 3:**
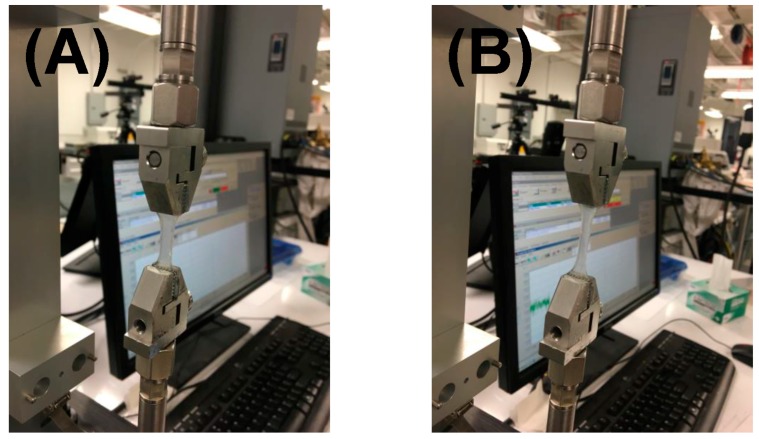
Mechanical testing to measure stiffness moduli of the hydrogels. Shown in (**A**) are the gels during the beginning of the experiment; in (**B**), the load is being applied and the gels appear stretched.

Average elastic modulus of the gels was about 117 ± 23 kPa, which implied that these hydrogels would maintain structural fidelity in vivo (Figure 3), as they appeared to be stiffer than other hydrogels commonly used for tissue engineering [24,35] and transplant applications [36,37]. This enhanced mechanical stiffness of these alginate–gelatin hydrogels poses several advantages. First, because of this enhanced stiffness, MSCs that are initially seeded atop the gels (in vitro), may migrate within the gels by durotaxis [38,39]. In addition, these stiff gels when implanted in vivo (laden with cells) could pose a relatively steep stiffness gradient compared to the neighbouring stomach tissue (stiffness of ~2 kPa [40]), which may initially promote migration by durotaxis of the stomach tissue cells towards the gels. Along with stomach tissue cell migration and fluid retention within the hydrogel scaffold, this should in turn enable loosening of the stiff structure and gradually promote release of cells (MSCs) and allow their migration from within the scaffold to the tissue.

### 3.5. Biocompatibility

The mouse MSCs used in this study reportedly possess a strong capacity to expand [41]. This was further confirmed using FACS analysis. During FACS analysis, the MSC cell culture extract after 72 h showed a 32% proliferating cell subpopulation (in tissue culture plastic) versus only 0.3% for nonstained controls (Supplementary Figure S1). This proved the high expansion capability of these mouse MSCs. 

**Figure 4:**
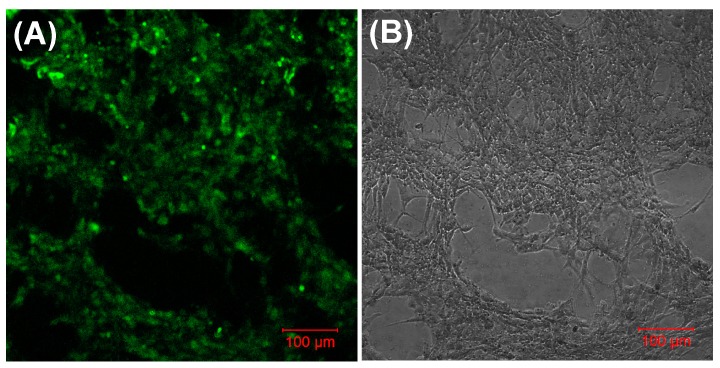
Cyto-compatibility of the poly-l-lysine-coated hydrogels. Shown in (**A**) are mouse mesenchymal stem cells (MSCs), prestained with PKH-67, growing uniformly across the gel; in (**B**), a brightfield image of the same is shown.

The in-vitro passaged and stabilized MSCs (~6th passage) were seeded atop these scaffolds in a density of 50,000 cells/mL initially. These cell–gel sheets were cultured in complete growth medium for at least 48 h, after which they were analyzed (Figure 4). Mouse MSCs grew uniformly across the entire surface of the gel on which the cells were initially seeded (Figure 4), and revealed an estimated average cell surface density of 1,407,185 ± 44 cells per square cm, calculated using Image J (NIH). However, when a z-scan was conducted over the entire thickness of the cell–gel constructs, maximum cell density was noted in the middle of the constructs (Supplementary Figure S2). This issue can be overcome using a cell bioprinting approach, wherein a homogenous layer-by-layer cell density can be easily achieved [42]. Only viable cell–gel constructs were placed atop luminal surfaces of mouse stomach tissue explants.

### 3.6. Delivery of Mouse MSCs from Gels to Stomach Tissue

**Figure 5:**
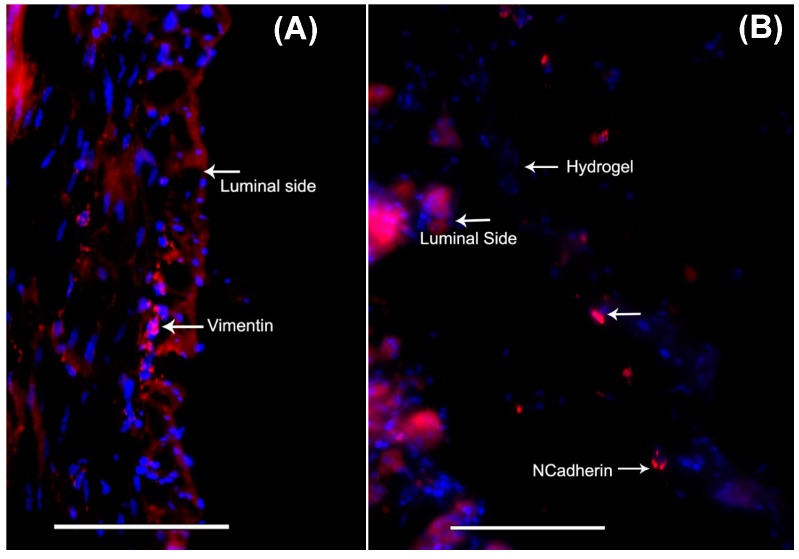
When the mouse-MSC-seeded gels were placed atop the stomach lumen, the cells migrated from the gels to the luminal tissues, as shown by positively stained cells for the stem cell markers, (**A**) vimentin and (**B**) *N*-cadherin. Scale bar in both images denotes 100 μm.

**Figure 6:**
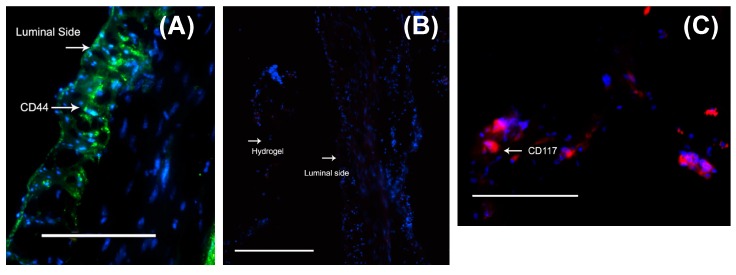
When the mouse-MSC-seeded gels were placed atop the stomach lumen, the cells migrated from the gels to the luminal tissues, as shown by positively stained cells for the MSC marker, CD44 (**A**) but not the negative marker, CD45 (**B**). Some of the migrated MSCs did exhibit CD117-expressing (ICC) cells (**C**). Scale bar in all images denotes 100 μm.

MSC migration within the mouse stomach was probed using antibodies against vimentin and *N*-cadherin, both of which are commonly used markers for stem cells [43,44,45]. Interestingly, mouse stomachs that received cell–gel constructs showed the presence of cells that stained positive using both markers, vimentin (Figure 5A) and *N*-cadherin (Figure 5B, during processing the gel got detached from the stomach tissue section). Control stomach explants, which received only gels or none, did not reveal such results. This proves that MSCs can be successfully delivered from the gels to the stomach tissue explants, and that the delivered cells were able to penetrate some substantial distance within the width of the stomach tissue explants used for the experiments. Although it is not feasible to estimate the penetration depth of the MSCs within the stomach tissue, acquired images revealed an average distance of at least 150 ± 50 μm from the stomach lumen surface up to which the MSCs had migrated in less than 48 h. Although *N*-cadherin is not an absolute marker for stemness, vimentin is frequently used for detecting the presence of MSCs [46]. In addition, we confirmed the uptake of MSCs within stomach tissue by CD44 staining [47] (Figure 6A). On the other hand, these cells did not show any staining with the negative marker CD45 [47] (Figure 6B). Furthermore, some of these MSCs did exhibit CD117-expressing (ICC) cells [48,49], which are shown in Figure 6C.

Given the multilayered structure of the stomach wall with the innermost mucosa, to the outermost smooth muscle layers, all of which contain ubiquitously high densities of ICC [12,50,51], it would be extremely beneficial to release a physiologically relevant number of MSCs in combination with the desired growth factors in a layer-by-layer approach from the bioengineered scaffold to target regeneration of the ICCs and other cell types in the stomach wall. Thus, our future goal is to 3D-bioprint the hydrogel scaffold with cells and growth factors. The main ICC focus is in the smooth muscle layer, particularly in circular and near myenteric plexus [12]. With the combination of layer-by-layer bioprinting of the scaffold and applying it for a longer period in vivo (2–4 weeks), we anticipate that MSCs can be delivered to the innermost layers of the stomach tissue. 

## 4. Discussion

Loss of ICC cells might be an underlying cause for the development of gastroparesis leading to symptoms such as nausea, vomiting, early satiety, postprandial fullness, bloating and abdominal pain [1]. Because of limited pharmacological options, surgery including gastric resections has been required with accompanying degrees of morbidity [1]. The limited capacity for food intake after this procedure creates a need for regenerating the stomach [2]. Therefore, using principles of tissue engineering, including isolated cells combined with appropriate biomaterials, can lead to the formation of a tissue-engineered stomach in vivo [2]. Cell-based approaches utilizing gastric–epithelial–organoid units seeded on polymeric scaffolds have been successful in stomach wall repair [2]. These results, along with more recent reports [2,52], emphasized the need for cell-based therapies for stomach tissue engineering, and may improve our understanding of the beneficial effect of ICCs by regenerative stem cell-based therapies in gastrointestinal complications of diabetes. However, the isolation of specialized units, such as the gastric–epithelial–organoid units, is technically challenging [2]. Therefore, a much more feasible alternative would be to deliver stem cells via a biomaterial scaffold for regenerating the stomach wall [8,10,53,54]. Recently, it was shown that gastric stem cells isolated from younger mice, when transplanted into sites of injury within the stomachs of older mice, resulted in accelerated repair [55]. This study, in addition to existing literature, highlights the benefits of stem cell transplantation as a glimpse into future treatment strategies for the healing of the motility-compromised stomach. Our study demonstrated that MSCs can be delivered via an alginate–gelatin scaffold to stomach tissue explants in vitro. 

The possibility of using bone marrow and other non-gut-derived murine MSCs for in-vivo immunosuppression after allogeneic transplantation is well established [8]. ICCs originate from mesenchymal precursors [54], and a number of animal studies have revealed the plasticity and regenerative capacity of ICCs in neonatal and adult ICC-damaged models, wherein it was demonstrated that the role of MSCs is particularly prevalent [56]. From recent observations that bone marrow (BM)-derived mesenchymal stem cells are capable of differentiating into osteoblasts, adipocytes, chondrocytes and myocytes [57], there is a strong possibility that BM-derived stem cells could also differentiate into ICCs. Earlier, it has been demonstrated that allotransplantation of ICCs into the myenteric plexus of the small intestine successfully induced distinct c-Kit^+^-congenitally deficient in myenteric ICC (ICC–MY) networks and rhythmic pacemaker activity [58]. Recently, a group performed BM transplantations to *W*/*W*_V_ mutant mice (congenitally deficient in myenteric ICC; ICC–MY) through a similar method to that employed in clinical circumstances [58]. Despite successful colonization, this method is not clinically feasible, owing to lack of clinical experience of its administration and the fact that these BMs cannot develop into intact and functionally mature ICCs under current conditions. Clinically, BM transplantation still presents an extremely high risk of life-threatening complications. This study may signify a substantial first step towards an innovative remedial cure for debilitating GI motility disorders by promising to restore damaged or interrupted ICC networks using this method. 

This study showed that it is possible to deliver MSC via an alginate–gelatin scaffold to stomach tissue explants in vitro. The feasibility of using bone marrow and other non-gut-derived murine MSCs for in-vivo immunosuppression after allogeneic transplantation is well known [8]. MSCs, when cultured in direct contact with other differentiated cell types or their cell-secreted extracellular matrix, can differentiate into the latter cell type [59]. So, if we could possibly deliver MSCs via biomaterial scaffolds to the stomach in vivo, we can then test their ability to restore levels of not only ICCs but also nNOS-expressing enteric neurons to heal the diseased stomach wall. In this study, we observed that the MSCs not only penetrated through the epithelium of the lumen into the intramuscular ICC region of the explant of the mouse stomach, but also showed possible signs of differentiation towards an ICC phenotype. 

Alginate-based biomaterials are extremely biocompatible and have been utilized as drug-delivery systems and cell carriers for tissue engineering [60]. Tuning the basic structure and properties of alginate such as biodegradability, stiffness, gelation and cell affinity can be achieved through combination with other biomaterials, immobilization of specific ligands such as peptide and sugar molecules, and physical or chemical crosslinking [60]. On the other hand, gelatin is favored in cell culture on account of its biodegradability, biocompatibility in vivo due to the presence of an RGD (Arg–Gly–Asp) sequence, and its commercial availability at low cost [61]. When combined in a single scaffold, alginate–gelatin hydrogels can confer both biocompatibility and mechanical rigidity at the same time and are thus popular candidates for tissue engineering applications [62]. Alginate gels can also be engineered as microgels [20,63], leaving open the possibility of being able to deliver MSCs encapsulated in such vehicles for treatment of GP in the future. Such microgels can be sustained within host tissues for longer-than-usual durations [64]. 

While studies have reported the isolation of stomach epithelium organoid units for tissue engineering of the stomach [2,5], this simple approach of delivering isolated MSCs from a scaffold is unique and is a simpler and more feasible alternative. Delivering stem cells for tissue engineering of the stomach has already proven to be promising [45]. Furthermore, if we incorporate the possibility of 3D bioprinting layer-by-layer of such scaffolds with cells, higher cell density for delivery can be achieved, as well as potent growth factors that can be delivered along with the cells for promoting their differentiation into ICCs or enteric neurons.

## 5. Conclusions

The results from this study imply that stem cells such as MSCs can be delivered from a biomaterial scaffold to stomach tissues, and this approach may be applied in vivo to help restore gastric function in GP. Thus, in the future, we hope to fabricate and characterize a tissue-engineered ‘gastric patch’ by 3D bioprinting of MSC–gel constructs containing selected growth factors to assess the outcomes of grafting the ‘gastric patch’ onto the luminal surfaces of the diabetic gastroparesis stomach wall in vivo, that includes the survival, adhesion and proliferation rates of the MSCs, the regeneration of ICCs and enteric neurons, and other physiological improvements in the stomach wall. 

The successful outcomes from this study are particularly significant in their ability to potentially study and impact other related gastric pathologies as well. Moreover, the cell–gel therapy can be administered to the stomach/GI tract in vivo during the course of a routine endoscopy procedure in humans, in the future as an envisioned and ideal clinical translation. The application of MSCs for possible regeneration of ICCs and other cells in the stomach wall will also inform and guide efforts in establishing new protocols that can be utilized to therapeutically intervene numerous other gastric pathologies.

## Supplementary Materials

The following are available online at http://www.mdpi.com/2306-5354/5/1/12/s1.

Supplementary File 1
