# Supplementary material for: Delivery of Mesenchymal Stem Cells from Gelatin–Alginate Hydrogels to Stomach Lumen for Treatment of Gastroparesis
Source: Bioengineering (Basel). 2018 Feb 7;5(1):12. doi: 10.3390/bioengineering5010012 (PMC5874878; doi:10.3390/bioengineering5010012)
Supplement: Supplementary File 1 [file bioengineering-05-00012-s001.docx]

**Supplementary Figure 1.** FACS analysis to confirm cell proliferation of mouse MSC cultured in tissue culture treated plastic wells (for ~72 hours). Left: unstained cells (controls), Right: Stained cells.

**Supplementary Figure 2.** Confocal Imaging of cells in gels counterstained for a. Actin, b. DAPI (nucleus) and c. merged (actin and DAPI). In d. Shown is a z-scan where maximum cell density is detected at the center of the gel.
